# Calciphylaxis, beware the ophthalmic mimic: A case series 

**DOI:** 10.5414/CNCS111088

**Published:** 2023-12-12

**Authors:** Chelsea Guymer, Sadia Jahan, Bobak Bahrami, David Sia, Bee Qung Tan, Stephen McDonald, Sumu Simon

**Affiliations:** 1 Department of Ophthalmology, and; 2 Central and Northern Adelaide Renal and Transplantation Services, Royal Adelaide Hospital, Adelaide, South Australia, Australia

**Keywords:** ophthalmic calciphylaxis, arteritic ischemic optic neuropathy, central retinal artery occlusion, kidney failure

## Abstract

Purpose: We present two atypical cases of calciphylaxis presenting with ocular ischemic pathology – both without the hallmark cutaneous manifestations – to raise awareness of this rare yet highly disabling condition. Observations: We report two cases of ophthalmic calciphylaxis presenting as (1) anterior ischemic optic neuropathy (AION) and cilioretinal artery occlusion in a 76-year-old woman with pre-dialysis kidney failure, and (2) AION with contralateral central retinal artery occlusion (CRAO) in a 44-year-old man on hemodialysis. Conclusion and importance: These cases highlight the need for judicious clinical suspicion of calciphylaxis in patients with kidney failure, presenting with microvascular ischemic ophthalmic pathology such as AION or CRAO. Confirmation with temporal artery biopsy is essential to direct targeted individualized multi-disciplinary treatment of calciphylaxis and avoid unnecessary steroid exposure in cases masquerading as giant cell arteritis (GCA).

## Introduction 

Calciphylaxis (calcific uremic arteriolopathy (CUA)) is a rare but highly morbid thrombogenic microvascular condition mostly seen in patients with kidney failure, with a reported incidence of 1 – 4% [20]. Other reported risk associations include female sex, White race, diabetes mellitus, obesity, autoimmune disease, thrombophilic disorders, liver disease, hypoalbuminemia and increased serum aluminum levels, hypercalcemia, hyperphosphatemia, hyperparathyroidism, adynamic bone disease, increased dialysis vintage, unmet dialysis adequacy parameters, high dialysate calcium bath, vitamin K antagonist (warfarin) therapy, malabsorption disorders, calcium supplements, calcium-based phosphate binders, vitamin D analogues, iron therapy, corticosteroids, subcutaneous injections, and POEMS (polyneuropathy, organomegaly, endocrinopathy, M-protein, and skin changes) syndrome [1, 2, 3, 4]. 

Cutaneous manifestations are the hallmark feature although other vessels can be affected. Calciphylaxis presents with solitary or multiple severely painful cutaneous or subcutaneous lesions (reticulate purpura, indurated plaques, or nodules) involving areas of adiposity that rapidly progress to ulcers with black eschar [1, 2, 5]. Wound infection and sepsis commonly ensue, and calciphylaxis is associated with high morbidity and mortality [3, 5]. Such typical skin lesions were absent in our reported cases, which instead presented with ophthalmic sequelae of microvascular occlusion and ischemic insult to the optic nerve and retina. 

Other organs known to be infrequently affected by calciphylaxis include skeletal muscle, intestines, mesentery, heart, penis, pancreas and lungs [1, 5]. Prognosis is poor, with reported mortality rates of up to 30% at 6 months and between 40 and 80% at 12 months [1, 6, 7]. 

Ocular calciphylaxis is exceedingly rare and can mimic giant cell arteritis (GCA). Calciphylaxis-associated non-arteritic AION has been reported in 12 cases in the literature to date [8, 9, 10, 11, 12, 13, 14, 15, 16, 17, 18, 19, 20, 21]. Even rarer, retinal ischemia secondary to calciphylaxis has been reported in only 6 case reports [6, 21, 22, 23, 24, 25]. One case described calciphylaxis-induced concurrent ischemic optic neuropathy, crystalline retinopathy, and ocular ischemic syndrome, with prior cutaneous involvement [22]. Most reported cases had evidence of concurrent or prior cutaneous calciphylaxis at the time of ocular involvement (Table 1) [6, 7, 8, 9, 10, 11, 12, 13, 14, 15, 16, 17, 18, 19, 20, 21, 22, 23, 24, 25]. 

## Case histories 

### Case 1 

A 76-year-old female presented with a 4-week history of transient “washed out” color vision and inferior subjective visual field obscuration affecting her left eye. She denied headaches, jaw claudication, or scalp tenderness. Ocular history was significant for moderate bilateral non-proliferative diabetic retinopathy (NPDR) with right vitreomacular traction and left epiretinal membrane with mild macular schisis. She had previously been treated with right scatter photocoagulation. Her medical history was notable for pre-dialysis kidney failure, type 2 diabetes mellitus, hypertension, and hyperlipidemia. 

**Figure 1:**
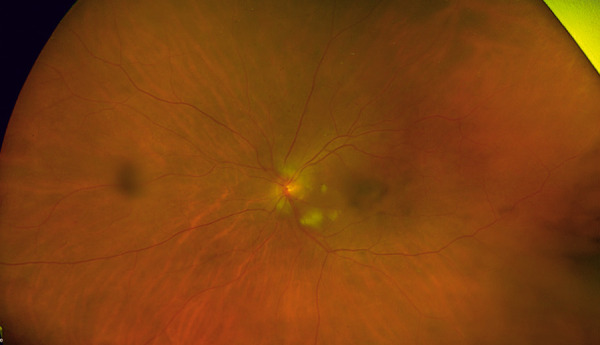
Color fundus photo of the left eye showing optic nerve head swelling, cotton wool spots along the cilioretinal artery distribution, and background moderate non-proliferative diabetic retinopathy.

**Figure 2:**
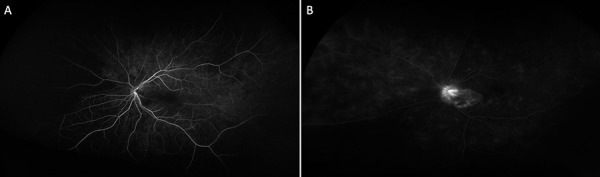
Fundus fluorescein angiography (FFA) at (A) 17 seconds showing nasal choroidal non-perfusion and (B) 5 minutes, showing disc leakage and late staining at the cilioretinal artery distribution.

Visual acuity was count fingers (CF) in the left eye and 6/30-1 in her right eye, with a dense left relative afferent pupillary defect (RAPD). She had advanced cataracts bilaterally, and fundal examination demonstrated left optic nerve head swelling, cotton wool spots in the cilioretinal artery distribution, and background bilateral moderate NPDR (Figure 1). Fundus fluorescein angiography (FFA) revealed nasal choroidal non-perfusion, disc leak, and cilioretinal artery occlusion (Figure 2). Her blood pressure was 163/72 mmHg, and bilaterally thickened tortuous non-tender temporal arteries were palpated. She had no cutaneous lesions. 

Investigations showed an elevated erythrocyte sedimentation rate (ESR) > 120 mm/h with a normal C-reactive protein (CRP) and platelet count (4.8 mg/L and 288 × 10 ^9^ /L, respectively). Calcium levels were normal (2.1 mmol/L), phosphate elevated (2.97 mmol/L, range: 0.7 – 1.5 mmol/L), creatinine 577 μmol/L, and estimated glomerular filtration rate (eGFR) of 6 mL/min/1.73m^2^, which were consistent with kidney failure. She also had secondary hyperparathyroidism, with a parathyroid hormone (PTH) level at 55.1 pmol/L (range: 0.8 – 5.5 pmol/L). There was no evidence of acute cortical ischemia nor significant cervical or intracranial arterial disease on computed tomography scan. Duplex carotid ultrasound showed no significant stenosis. A non-contrast MRI head was unremarkable. Left temporal artery duplex ultrasound showed tortuous small-caliber temporal arteries with heavy calcification of the main trunk extending into the frontal and parietal branches. 

**Figure 3:**
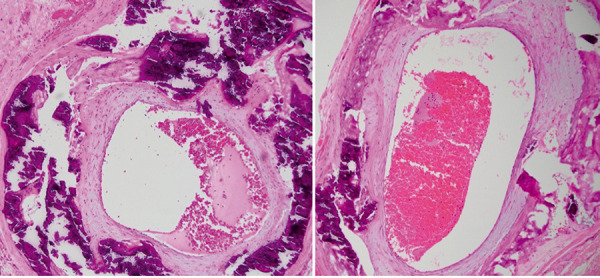
Temporal artery biopsy showing severe calcification and marked intimal thickening within the media and outer intima.

With a presumptive diagnosis of AION and given that giant cell arteritis (GCA) could not be excluded, intravenous methylprednisolone was commenced to preserve vision. Initial left temporal artery biopsy (TAB) was equivocal for calciphylaxis, demonstrating marked arterial medial calcification but no inflammatory activity. Repeat TAB on the right confirmed the diagnosis of calciphylaxis, showing severe circumferential arterial calcification and marked intimal thickening, and no convincing evidence of vasculitis (Figure 3). Based on these findings, steroid therapy was promptly tapered and discontinued, hemodialysis was initiated, cholecalciferol supplementation ceased, and lanthanum (non-calcium-based phosphate binder) commenced. Her left eye visual acuity (VA) remained stable (CF). 

### Case 2 

A 44-year-old man presented with acute onset left painless monocular vision loss. His medical history was significant for anti-glomerular basement membrane (anti-GBM) disease complicated by kidney failure. He was receiving hemodialysis for a total of 5 years, with a background of two prior living renal transplants. Severe tertiary hyperparathyroidism had been present for some time, refractory to large doses of cinacalcet. He had well-controlled hypertension, atrial flutter on warfarin for 18 months, and was an active smoker. No prior ocular history was declared. 

**Figure 4:**
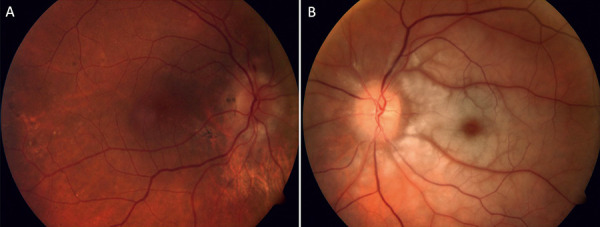
Color fundus photos demonstrating right circumferential (A) optic disc swelling with small superior disc flame hemorrhage and left (B) inferior pallid disc swelling, widespread retinal pallor with a cherry-red macula suggestive of central retinal artery occlusion.

Visual acuity was hand movements in the left eye, compared to 6/9 in his right eye, with a dense left RAPD. Anterior segments were unremarkable, with fundoscopy demonstrating left inferior pallid disc swelling with a cherry-red macula suggestive of central retinal artery occlusion (CRAO) (Figure 4B). Incidentally, circumferential right optic disc swelling was noted on the contralateral side (Figure 4A). Blood pressure was 120/77 mmHg, and bilaterally thickened non-tender temporal arteries were palpated. No cutaneous lesions were present. 

**Figure 5:**
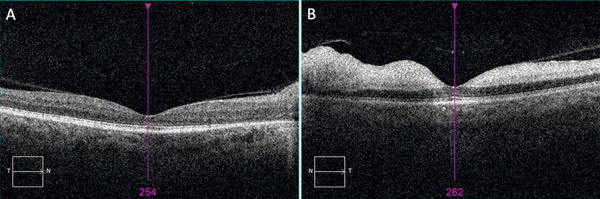
Spectral domain optical coherence tomography (SD-OCT) scans of the macula, demonstrating right (A) nasal Retinal nerve fibre layer (RNFL) thickening not involving the macula with normal foveal contour and (B) left macula hyper-reflectivity with signal increase and loss of distinction of the inner retinal layers.

**Figure 6:**
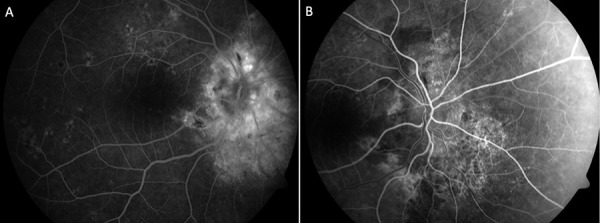
Fundus fluorescein angiography (FA) delayed choroidal filling with disc hyper-fluorescence and late patchy venous staining in the right eye (A) and delayed choroidal filling and delayed arteriovenous transit time in the left eye (B).

Ancillary testing with Spectral domain optical coherence tomography (SD-OCT) showed marked circumferential thickening of the right retinal nerve fibre layer (RNFL) with macular sparing, and left macula hyper-reflectivity with signal increase and loss of distinction of the inner retinal layers (Figure 5). HVF 30-2 demonstrated a right enlarged blind spot and complete loss of visual field in his left eye. FFA demonstrated delayed choroidal filling with disc hyper-fluorescence and late patchy venous staining in the right eye (Figure 6A). In the left eye, there was significant delayed choroidal filling and delayed arteriovenous transit time (Figure 6B). These findings were indicative of the left AION and right CRAO. 

Pathology showed an elevated ESR and CRP rising to peaks of 85 mm/h and 22.1 mg/L, respectively. Platelet count was mildly suppressed at 112 × 10^9^/L. Serum calcium and ionized calcium were normal (2.30 mmol/L and 1.11 mmol/L, respectively), phosphate was elevated (2.19 mmol/L), and parathyroid hormone was substantially elevated (100 pmol/L). Vasculitic screen was unremarkable (negative antineutrophil cytoplasmic antibodies (ANCA), antinuclear antibody (ANA), angiotensin-converting enzyme (ACE), rheumatoid factor (RF), normal C3 and C4). His thrombophilia screen was also unremarkable (normal antithrombin level, protein S level, mildly suppressed protein C level consistent with known warfarin therapy, negative lupus anticoagulant and β-2-glycoprotein antibody). 

CT brain and CT angiogram showed no evidence of other acute ischemic intracranial pathology, and duplex carotid ultrasound demonstrated bilateral < 50% internal carotid artery stenosis. MRI brain and orbits was not tolerated despite oral sedation due to claustrophobia. Ultrasound of the temporal arteries showed extensive calcific walls and marked tortuosity. Parathyroid ultrasound and nuclear medicine scanning showed parathyroid hyperplasia. Echocardiogram excluded a cardiac thromboembolic source. 

**Figure 7:**
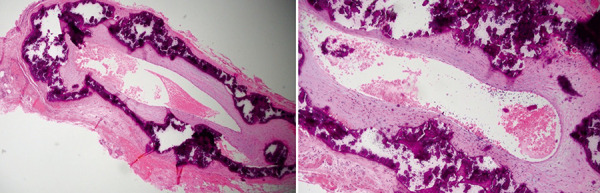
Temporal artery biopsy showing extensive circumferential calcification of the media with some associated intimal hyperplasia.

Given his younger age and known kidney failure, left TAB (Figure 7) was performed and confirmed calciphylaxis with arterial compromise as the unifying cause of his left CRAO and right AION. As he had developed tertiary hyperparathyroidism refractory to large doses of cinacalcet, he underwent an emergency subtotal parathyroidectomy with histology confirming widespread nodular parathyroid hyperplasia. Post-operatively he was stabilized with a calcium chloride infusion, which was quickly weaned down to oral calcium carbonate and weaning doses of calcium effervescent and calcitriol until calcium levels had stabilized. He was counselled regarding his guarded visual prognosis. Warfarin was ceased, and he was switched to low molecular weight heparin. We elected not to administer sodium thiosulfate as there were no painful cutaneous lesions, nor did he receive hyperbaric oxygen therapy. 

## Discussion 

Calciphylaxis is defined as a metastatic calcification of microvasculature with eventual thrombosis, vessel occlusion, and infarction of affected tissue. Known to predominantly affect arteries, this obliterative vasculopathy can also occur in small arteries and venules, and even capillaries [5]. Whilst incompletely understood, it is appreciated that elevated levels of vitamin D, hyperparathyroidism, hyperphosphatemia, and oxidized low-density lipoprotein can trigger the differentiation of vascular smooth muscle cells to produce bone matrix proteins, which precipitate microvascular calcification [1, 3, 4, 5, 15]. Interestingly, the role of local and systemic hypercoagulable states, including systemic thrombophilia’s, is gaining increasing attention in the development of calciphylaxis with the propagation of thrombi in ischemic vessels [3, 4]. This is further supported by histopathologic findings demonstrating that most patients with calciphylaxis-induced thrombosis had no inflammatory infiltrates suggestive of a vasculitic process [4]. This may in part explain why calciphylaxis can also arise in patients with normal kidney function (known as nonuremic calciphylaxis). 

A high clinical index of suspicion and histopathological confirmation of calciphylaxis is key for definitive diagnosis, particularly in patients with known kidney failure. Whilst hyperphosphatemia, hypercalcemia, and hypo/hyperparathyroidism are commonly found in those with calciphylaxis, many patients can have normal PTH concentrations [2, 5]. Calciphylaxis-associated AION, albeit rare, can masquerade as GCA, particularly in the elderly, yet tends to lack the symptoms of jaw claudication, scalp tenderness, and temporal headache typical of GCA. However, the lack of these symptoms does not assist in differentiating the diagnosis, as up to 20% of patients with GCA do not exhibit these typical systemic symptoms [21]. ESR is typically elevated in both calciphylaxis-associated AION and GCA [8]. Thus, TAB is essential in delineating calciphylaxis from GCA. TAB in GCA typically demonstrates features of chronic granulomatous inflammation including panarteritis with mononuclear cell infiltrates penetrating all layers of the arterial wall, with associated disruption of the elastic layer architecture and intimal hyperplasia [14, 15]. Whist focal calcification can be present, it is often secondary to atherosclerosis and is not a predominant feature [15]. In contrast, calciphylaxis usually demonstrates minimal inflammation with extensive medial artery calcification affecting the entire arteriolar circumference, intimal proliferation, and endovascular fibrosis of small-medium blood vessels [5, 15]. 

The management of calciphylaxis requires a multimodal and multidisciplinary approach, individualized to the patient’s clinical presentation. Given the rarity of this disease, the evidence base for calciphylaxis treatment is limited to retrospective data and clinical experience [3]. Treatment may involve correcting hyperphosphatemia (e.g., intensification of dialysis sessions, dietary phosphate restriction, use of non-calcium phosphate binders such as sevelamer or lanthanum, hypercalcemia (e.g., cease calcium-based phosphate binders, reduce calcium concentration in dialysis bath), and hyperparathyroidism (e.g., cinacalcet therapy, surgical parathyroidectomy in medically refractory cases) [2, 3, 4]. Supplemental vitamin D and warfarin therapy should be ceased, with consideration of alternative non-vitamin K-dependent anticoagulant therapy (such as apixaban, or heparin) [2, 3, 4]. Conversion from peritoneal dialysis to hemodialysis should also be considered to help improve clearances. Sodium thiosulfate, a calcium chelator, can be used to promote the decalcification of blood vessels [1, 2, 5]. As an antioxidant, sodium thiosulfate may also neutralize reactive oxygen species, thereby reducing inflammation, thrombosis, and vasoconstriction [2, 3, 5]. Hyperbaric oxygen can be useful in aiding wound healing in patients with cutaneous disease but is also an important option for patients who are not surgical candidates for, or have refractory disease following, parathyroidectomy, and those who do not have secondary or tertiary hyperparathyroidism [5]. Additionally, hyperbaric oxygen may be of benefit in patients with CRAO if instigated within 24 hours of the onset of visual symptoms [26]. 

## Conclusion 

Whilst appreciated to be a cutaneous disease, rarely, calciphylaxis can manifest as ischemic ophthalmic microvascular disease such as AION, CRAO, or ocular ischemic syndrome. Calciphylaxis should be suspected as a differential for patients with kidney failure presenting with AION or CRAO, particularly in younger patients or those lacking the typical systemic symptoms of GCA. Judicious clinical evaluation and histopathological confirmation with a TAB is essential to exclude this life-threatening condition and dictate further management. 

The successful treatment of ophthalmic calciphylaxis requires a skilled multidisciplinary effort, tailored to the patient, with close collaboration between physicians and ophthalmologists. Strategies are primarily aimed at stopping the calcification, inhibiting thrombosis, and, in the case of cutaneous disease, wound management and pain control. 

## Consent 

Written patient consent was obtained in both cases for the publication of this case series. 

## Funding 

No funding was received for the production of this manuscript. 

## Conflict of interest 

The authors have no conflict of interest to declare. 

**Table 1:** Summary of literature describing ocular calciphylaxis.

Case	Sex/Age	Symptoms	Ocular disease	Other sites involved	Renal disease	Laboratory results	Cutaneous/temporal artery biopsy	Treatment
Case 1: Awwad et al. (2010) [12]	Male, 75 years	Blurred vision in left eye Painful dusky skin lesions over bilateral calves	Left AION	Skin: dusky purpuric plaques (confirmed calciphylaxis on skin biopsy) on bilateral calves	Type 2 diabetes mellitus (on insulin) ESRD on peritoneal dialysis Coronary artery disease (on warfarin) Hypertension	ESR 106 mm/h Calcium 7.7 mg/dL Phosphorus 9.5 mg/dL Creatinine 12.3 mg/mL	Diffuse and extensive circumferential calcification at the internal elastic lamina and media, minimal inflammation, no granuloma	Prednisolone 80 mg prior to diagnosis of calciphylaxis *Further treatment not detailed in report*
Case 2: Chehade et al. (2020) [16]	Male, 67 years	Painless left visual decline over 24 hours Fluid overload	Left AION and BRAO	Heart: nodular mass on posterior mitral valve leaflet – fibroelastoma with calcification *No cutaneous involvement*	Type I diabetes mellitus Ischemic heart disease NSTEMI and coronary artery bypass graft Hypertension Peripheral vascular disease Bilateral critical limb ischemia with below-knee amputation ESRD	ESR 49 mm/h CRP 26 mg/L Urea 29.6 mmol/L Creatinine 415 μmol/L Calcium 2.01 mmol/L Platelets × 10^9^/L	Extensive circumferential calcification, intimal swelling and luminal narrowing, occasional multinucleate giant cells, no background granulomatous inflammation	IV methylprednisolone 3 days then oral prednisolone whilst awaiting TAB Hemodialysis commenced Cinacalcet
Case 3: Cherayil et al. (2021) [22]	Female, 72 years	Bilateral vision loss Preceding painful gluteal nodules – biopsy-confirming calcinosis cutis	Bilateral ischemic optic neuropathy and ocular ischemic syndrome (R > L) Left crystalline maculopathy	Skin: calcinosis cutis of bilateral gluteal muscles	ESRD: Membranous glomerulonephritis Failed renal transplant Peritoneal dialysis Type 2 diabetes Hypertension with systolic heart failure	Serum correct calcium 9.4 mg/dL Phosphate 12.8 mg/dL Calcium-phosphate product 120.3 mg^2^/dL^2^ PTH 1,365 pg/mL Creatinine 16.70 mg/dL Serum oxalate 23.0 μmol/L	TAB not performed Calciphylaxis diagnosed on skin biopsy (calcinosis cutis) and clinical findings	IV sodium thiosulfate through hemodialysis
Case 4: Danset et al. (2021) [25]	Male, 67 years	Painful glans penis, painful skin lesions on bilateral thighs and necrotic calf ulcer	Left CRAO	Skin: glans penis gangrenous ulceration, bilateral panniculitis of the thighs and calf ulcer	Type 2 diabetes Hypertension Arrhythmic cardiomyopathy Dyslipidemia Obesity ESRD (IgA glomerulonephritis), on peritoneal dialysis	PTH 294 ng/L Normal calcium phosphate 2.23 mmol/L	Not performed Calciphylaxis diagnosed on skin biopsy	Hemodialysis IV sodium thiosulfate Rheopheresis Discontinuation of fluindione and vitamin D
Case 5: Farooqui et al. (2019) [9]	Male, 39 years	Sudden painless vision loss left eye	Left AION	Nil *No cutaneous involvement*	ESRD (glomerulonephritis), on hemodialysis	ESR 120 mm/h CRP 74.1 mg/L Negative vasculitis work-up Normal platelet count Serum phosphate 1.72 mmol/L Low total calcium 1.55 mmol/L Normal PTH 2.1 pmol/L	Calcification of the medial and severe luminal occlusion, no inflammatory findings, no giant cells	Short oral steroids with rapid taper after TAB IV sodium thiosulphate with hemodialysis
Case 6: Hanna et al. (2015) [23]	Male, 64 years	Severe abdominal pain, acute anuria, confusion Then 3 months later, sudden left eye blindness	Left ophthalmic artery occlusion	Skin: lower limb popular lesions (biopsy provide calcinosis) Genitalia: glans penis dry gangrene (biopsy-confirmed calciphylaxis), testicular microliths Renal: acute tubular necrosis, microliths	Hypertension Type 2 diabetes Congestive cardiac failure (systolic) Chronic renal disease stage 3 (diabetic glomerulosclerosis)	Serum creatinine 6 mL/min/1.73m^2^ (eGFR 6) Potassium 7.4 mmol/L Urea 178 mg/dL Corrected calcium 10.2 mg/dL Phosphorous 13.5 mg/dL Calcium phosphorous product 121.5 mg^2^/dL^2^ HIV/hepatitis serology negative ANA/ANCA negative PTH 612 pg/mL Negative vasculitis screen	Skin biopsy calcinosis	Hemodialysis IV piperacillin/tazobactam for penile gangrene Partial penectomy
Case 7: Hepschke et al. 2019 [21]	Male, 78 years	Sudden-onset painless reduced right vision, with preceding non-healing necrotic leg ulcers	Right CRAO and AION	Skin: non-healing necrotic leg ulcers	Hypertension Hyperlipidemia Ischemic heart disease Atrial fibrillation (on warfarin) CABG Aortic valve replacement Gout ESRD (on hemodialysis) Carotid and vertebral artery stenosis (80 – 99%)	Platelets 219 × 10^9^/L ESR 102 mm/h CRP 98 mg/L CT brain: diffuse calcification of vessels MRI brain: changes of chronic microvascular disease, no focal cerebral ischemia “borderline elevated calcium and phosphate” eGFR 13 mL/min/1.73 m^2^	TAB: focal myxoid degeneration and extensive concentric calcium deposition in the tunica media, no features of GCA	IV methylprednisolone whilst awaiting TAB Hyperbaric oxygen Hemodialysis with low calcium replacement fluids Cessation of vitamin D supplementation Warfarin ceased and changed to LMWH and aspirin
Case 8: Huerva et al. (2011) [13]	Female, 51 years	Bilateral sequential vision loss	Sequential bilateral AION	Skin: necrotic cutaneous lesions at the distal phalanges of right-hand fingers	ESRD (sclerosis tuberose and bilateral nephrectomy) – on hemodialysis Secondary hyperparathyroidism (subtotal parathyroidectomy)	Normal vasculitic screen (ANCA, ACA assays) Syphilis serology negative Normal albumin, vitamin B12, folic acid, immunoglobins and cryoglobulins ESR 70 mm/h	Skin biopsy: calcification in the wall of small artery TAB: large calcium deposits in the artery tunica media	IV methylprednisolone for 3 days then oral steroids for 2 months Hemodialysis Surgical debridement of skin lesions and hyperbaric oxygen
Case 9: Komurcu et al. (2016) [20]	Female, 43 years	Sudden-onset visual impairment	Bilateral AION	Skin: acral gangrene (bilateral hands and feet) Visceral ischemia (mesenteric, spleen, renal)	ESRD (on hemodialysis)	Normal ESR/CRP Negative vasculitis, autoimmune and infectious screen Serum calcium 8.8 mg/dL (normal) Phosphorus 12.5 mg/dL PTH 137 pg/mL	Thumb amputation: calcification, thrombi, obstructive endovascular fibrotic areas in the walls of arteries	IV methylprednisolone then oral steroids (up to 15 days) Pentoxifylline Acetylsalicylic acid Hemodialysis
Case 10: Korzets et al. (2004) [11]	Patient 1: Male, 77 years	Right eye vision loss	Right AION	Widespread arterial wall calcifications *No cutaneous involvement*	Hypertension Type 2 diabetes mellitus ESRD (on hemodialysis) Ischemic heart disease with previous myocardial infarction Peripheral vascular disease (toe amputation) Aortic valve replacement (on warfarin) Widespread arterial calcifications	Serum phosphorous 10 mg/dL Serum PTH 950 pg/mL	TAB: profound medial calcifications	IV methylprednisolone (ceased after 5 days)
	Patient 2: Female, 70 years	Bitemporal headaches with blurred vision in inferior right visual field	Right AION	Nil *No cutaneous involvement*	Hemodialysis IgG γ-monoclonal gammopathy of undetermined significance Atherosclerosis Myocardial infarction with CABG Peripheral vascular disease with left below-knee amputation Bilateral cataract surgery	Serum phosphorous 10 mg/dL PTH < 100 pg/mL	TAB: concentric medial calcification, with no features of arteritis	IV methylprednisolone (ceased after 4 days)
Case 11: Mishra et al. (2021) [11]	Female, 58 years	Bilateral reduced vision	Retinal arteriolar calcification with ischemic retinopathy	Nil *No cutaneous involvement*	ESRD (obstructive uropathy from staghorn calculi) Right percutaneous nephrolithotomy with stent Not on dialysis	Urea 123 mg/dL Creatinine 12.9 mg/dL Calcium 8.2 mg/dL Phosphate 7.3 mg/dL PTH 233.8 pg/mL Vitamin D3 26.96 ng/dL Calcium phosphate product 59.86 mg^2^/dL^2^ eGFR 26 mL/min/1.73m^2^	*Not reported*	Pan retinal photocoagulation
Case 12: Naysan et al. (2018) [6]	Male, 65 years	Poor vision bilaterally	Bilateral crystalline retinopathy with prior right CRAO	Skin: necrotic skin lesions of upper thigh (prior to ocular symptoms)	ESRD (obstructive nephropathy from nephrolithiasis, on hemodialysis)	Normal range corrected total serum calcium and phosphorus levels	Skin biopsy: medial arterial wall calcification and necrosis of the skin epithelium confirming calciphylaxis	*Not reported*
Case 13: Roverano et al. (2015) [8]	Female, 82 years	Sudden vision loss in left eye No headache, jaw claudication, proximal muscle weakness, weight loss	Ocular: AION	Nil *No cutaneous involvement*	Not reported (non-uremic calciphylaxis – no prior history of ESRD)	ESR 62 mm/h Urea 0.56 g/dL Serum creatinine 1.7 mg/dL Low calcium (8.39 mg%) and phosphorus (2.40 mg/dL) Normal urine calcium (74 mg/24h) Normal PTH (51.41 pg/mL) Autoimmune/vasculitic screen normal (anticardiolipins, lupus anticoagulant, ANCA, IgG and subtypes)	TAB: large calcium deposits in the artery’s tunica media and internal elastic lamina, luminal narrowing, no inflammation nor multinuclear giant cells	IV methylprednisolone then oral prednisolone (ceased upon TAB findings confirming calciphylaxis)
Case 14: Shah et al. (2012) [17]	Male 72 years	Sudden vision loss in left eye, then right eye 3 days later	Ocular: Bilateral sequential AION	Skin: widespread dusky skin plaques that became gangrenous (limbs and glans penis) – onset after ocular symptoms	ESRD (on peritoneal dialysis) Hypertension Diabetes mellitus Aortic valve replacement (aortic stenosis, on warfarin) Atrial fibrillation CCF Tricuspid regurgitation Pulmonary hypertension Left ventricular hypertrophy Diabetic retinopathy and nephropathy	BUN 69 mg/dL Creatinine 6.3 mg/dL GFR 9 mL/min/1.73 m^2^ Calcium 7.9 mg/dL Phosphorus 6.6 mg/dL Calcium phosphate product 51.48 mg/dL PTH 164 pg/mL	TAB: large coarse marked calcifications in the muscular artery with no evidence of vasculitis Skin biopsies: calciphylaxis, with prominent calcification of the small vessels in the subcutaneous fat with ischemic necrosis and ulceration of the overlying epidermis	IV steroids (ceased following TAB results) Hemodialysis commenced Calcium acetate stopped and replaced with sevelamer (non-calcium phosphate binder)
Case 15: Sivertsen et al. (2018) [19]	Female 72 years	Reduced vision left eye No headache, jaw claudication, or symptoms of polymyalgia rheumatica Prior painful leg ulcers with recurrent bacterial infections	Ocular: left AION	Skin: leg ulcers	HTN AF (on warfarin) Type 2 diabetes mellitus Rheumatoid arthritis No prior history of ESRD (non-uremic calciphylaxis)	ESR 57 mm/h CRP 28 mg/L Corrected calcium 2.56 mmol/L Phosphate 1.36 mmol/L Creatinine 33 μmol/L PTH 3.5 pmol/L	TAB: extensive medial and intimal calcification consistent with calciphylaxis, no features of arteritis	IV sodium thiosulfate

AF = atrial fibrillation; AION = arteritic ischemic optic neuropathy; ANA = antinuclear antibody; ANCA = antineutrophil cytoplasmic antibody; BRAO = branch retinal artery occlusion; BUN = blood urea nitrigen; CABG = coronary artery bypass graft; CCF = congestive cardiac failure; CRAO = central retinal artery occlusion; CRP = c-reactive protein; eGFR = estimated glomerular filtration rate; ESR = erythrocyte sedimentation rate; ESRD = end-stage renal disease; GCA = giant cell arteritis; GFR = glomerular filtration rate; HTN = hypertension; IV = intravenous; LMWH = low molecular weight heparin; PTH = parathyroid hormone; RAPD = relative afferent pupillary defect; TAB = temporal artery biopsy.
